# Towards an advanced cell-based *in vitro* glioma model system

**DOI:** 10.3934/genet.2018.2.91

**Published:** 2018-03-19

**Authors:** Valeriia Mikhailova, Valeriia Gulaia, Vladlena Tiasto, Stanislav Rybtsov, Margarita Yatsunskaya, Alexander Kagansky

**Affiliations:** 1 Center for Genomic and Regenerative Medicine, School of Biomedicine, Far Eastern Federal University, Vladivostok, Russian Federation; 2 Scottish Centre for Regenerative Medicine of the University of Edinburgh, Edinburgh, United Kingdom; 3 Federal Scientific Center of the East Asia Terrestrial Biodiversity FEB RAS 159, Stoletij Vladivostoku Avenue, 690022, Vladivostok, Russian Federation

**Keywords:** brain tumors, glioma, *in vitro* tumor model system, glioma model, neurospheres, glial cell lines, primary glial cell cultures, cancer stem cells

## Abstract

The modulation of tumor growth and development *in vitro* has always been one of the key factors in the research of the malignant transformation, including gliomas, prevalent and most deadly cancers of the brain. Indeed, cellular and molecular biology research employing *in vitro* model cell-based systems have great potential to advance both the mechanistic understanding and the treatment of human glial tumors, as it facilitates not only the understanding of glioma biology and its regulatory mechanisms Additionally they promise to afford the screening of the putative anti-tumor agents and alternative treatment approaches in a personalized manner, i.e. by virtue of using the patient-derived tumor material for such tests. However, in order to become reliable and representative, glioma model systems need to move towards including most inherent cancer features such as local hypoxia, specific genetic aberrations, native tumor microenvironment, and the three-dimensional extracellular matrix.

This review starts with a brief introduction on the general epidemiological and molecular characteristics of gliomas followed by an overview of the cell-based *in vitro* models currently used in glioma research. As a conclusion, we suggest approaches to move to innovative cell-based *in vitro* glioma models. We consider that main criteria for selecting these approaches should include the adequate resemblance to the key *in vivo* characteristics, robustness, cost-effectiveness and ease to use, as well as the amenability to high throughput handling to allow the standardized drug screening.

## 1. Introduction

Primary tumors of the central nervous system (CNS) comprise relatively rare oncological pathologies in comparison to other common neoplasms [1]. Nevertheless, the incidence and mortality of the patients diagnosed with the CNS cancer is continuously growing. According to the official statistics, the incidence of primary tumors of the central nervous system was 4.8 cases per 100,000 in 2015 (4.2 cases in 2010) in Russian Federation [2]. The 5-year survival rate ranges from 66% for younger (under 19 years old) people down to 5% for the older (75 years and older). In case of ependymomas and oligodendrogliomas, the 5-year survival is favorable: 85 and 81% patients aged 20–44, 69 and 45% patients aged 55–64. The prognosis is less favorable for multiform glioblastoma: 13% patients aged 20–44 and 1% patients aged between 55 and 64 survive for five years [2]. In Russian Federation, CNS tumors were second most common cause of mortality among all cancer patients aged 0–29 (18.9%) and male patients aged 30–39 (12.4%). The death rate for these diseases increased from 2006 to 2016 by 27.16% making it the deadliest category among all forms of cancer [2].

According to the Central Brain Tumor Registry of the United States (CBTRUS), in the United States the incidence rate of all primary malignant brain and other CNS tumors is 7.15 per 100,000 [3]. In 2010–2014, CNS tumors became the most common cancer among the 0–14 years patients and third most common cancer among the 15–39 years patients, according to the CBTRUS [3]. An estimated 23,830 new cases of primary malignant brain and other CNS tumors are expected to be diagnosed in the United States in 2018 [3]. The worldwide incidence rate in 2012 was 3.4 per 100,000. Primary malignant brain and other CNS tumors incidence rates were 3.9 cases per 100,000 males and 3.0 cases per 100,000 females. The incidence rates were higher in the developed countries (5.1 per 100,000) than in developing countries (3.0 per 100,000) [4].

The mortality rates for the primary CNS tumors in the USA among people aged 0–14 and 15–39 are similar, being 0.70 per 100,000 and of 0.95 per 100,000 respectively, thus putting CNS tumors among the most aggressive and fatal cancer types in the United States [3].

Glioma represents 24.7% of all primary brain tumors and 74.6% of all malignant tumors [5]. It is a broad term, combining all tumors arising from glial or neural precursor cells. The majority of them is characterized by diffuse infiltrative growth into the surrounding CNS parenchyma [6] and could be histopathologically distinguished as astrocytomas (including glioblastoma), oligodendrogliomas, oligoastrocytomas (mixed gliomas), and ependymomas, according to the 2007 World Health Organization (WHO) classification [7].

The 5-year gliomas survival rates vary from 53% for oligodendrogliomas and 30% for astrocytomas, to only 5% for glioblastomas [8]. Glioblastoma accounts for two-thirds of all glioma cases [9]. with the overall survival lower than 2 years and relapses occurring in almost 100 cases within 5–9 months after standard treatment [10]. For low-grade gliomas, the overall survival rate is substantially higher, being 16 years for oligodendroglioma patients and 8 years for astrocytoma patients [11]; nevertheless, relapses occur in approximately half of the cases [12].

The glioma incidence rate varies considerably across the world predominantly due to registering glioma cases based merely on radiological data or even anamnesis, as in some countries, despite missing the histological confirmation. To this end, in this review we decided to include only the incidence data registered based on histologically validated data, as it is done by CBTRUS, FBTDB, and ABTR registries. Therefore, the adjusted incidence rate per 100,000 for diffuse low-grade gliomas is 0.76 worldwide, 0.86 in Europe, and 0.83 in USA [13]. The 5-year survival estimated by RARECARE project is around 5 and 43% for high and low grade astrocytomas respectively, while for oligodendrogliomas this index is reaching 30 and 65% for high and low tumor grades, respectively [14].

## 2. Biological challenges

The unfavorable prognosis and the high frequency of glioma relapse are supposed to be associated with the presence of non-dividing cancer stem cells (CSCs) resistant to chemo- and radiotherapy due to the reduced cell cycle genes expression and the activation of the resistance genes to alkylating chemotherapy drugs [15],[16]. Surgical removal of the bulk tumor does not lead to the successful treatment especially for high grade gliomas as the complete resection cannot always be achieved because of the tumor frontier not being apparent, resulting in either residual tumor cells or the damage to adjacent vital zones of the brain. After surgical tumor removal, the remaining tumor cells, which were not destroyed by subsequent chemo- or radiotherapy, tend to restore the entire heterogeneous tumor structure within next six months causing the disease relapse accounting for the dramatic drop of the overall survival rates [17],[18].

Over the past decade, there has been an increasing awareness of the heterogeneous nature of the brain tumors and of its critical importance for the understanding and treatment of these diseases. Tumors from different patients with the same histology-based diagnosis, nevertheless have distinct genotype and malignant potential [19]. Most tumors are supposed to originate from a single clone with genomic aberrations that are present in all tumor cells [20]–[22]. With the disease progression, tumor cells initially possessing common initiating mutations start accumulating additional somatic mutations leading to highly heterogeneous cell populations. Averaging genotypes across the whole tumor may lead to rare albeit therapeutically relevant subpopulations containing quiescent stem cells evading cell cycle blockers [19]. Furthermore, cancer cells within one tumor have functional heterogeneity, and it has been suggested that limited proportion of cancer cells may possess tumor-initiating ability (i.e. cancer stem cells population, CSCs) [23],[24].

Currently, the hypothesis about the predominant definitive contribution of CSCs required for the initiation and growth of malignant tumors is the most widely accepted [25]. Indeed, in the normal organism, these are the stem cells, which are able to differentiate into all cell types and thereby reproduce the entire spectrum of cell characteristics in the relevant tissues, and hence responsible for the glioma recurrence. CSCs comprise a small tumor cells population possessing virtually unlimited proliferative potential, markers of normal stem cells and the ability to restore the entire tumor structure when implanted into immunodeficient mice [26]. The hypothesis regarding CSCs primary contribution to the initiation and the growth of the glial tumors is reinforced by their phenotypic variability, also characteristic of the normal stem cells, providing satisfactory explanation for the phenomenal gliomas chemo- and radiotherapy resistance. The main theory behind CSCs resistance is the presence of a quiescent, non-dividing population with the potential to produce all the cell types constituting the tumor [27]. Cycle arrest caused by the most chemotherapeutic agents does not lead to the elimination of this population, since CSCs predominantly rest in the quiescent condition (G0 phase) enabling them to evade the chemotherapeutic treatment [25]. An additional component affecting the acquisition of certain tumor cell properties are tumor microenvironment and the extracellular matrix defining the tumor niche. Tumor microenvironment includes non-tumor cells (stromal and immune system cells) that support the development and growth of tumor cells, protecting them from the immune response and creating special niche properties e.g. hypoxia, vessel growth, and high concentrations of growth factors [28],[29].

According to the popular theory, CSCs are the main source of heterogeneity in a growing tumor, giving raise to the numerous clones that differ in their driver mutations, transcriptome profiles and sensitivity to drugs [30],[31]. The isolation and characterization of glioma CSCs is complicated by the absence of universal phenotypic markers, but a number of candidate proteins have been proposed for its role: СD133 [32], CD44 [33], CD49f [34], Musashi-1 [35], Nestin [36], Nanog [37], Oct4 [38] and Sox2 [39]. There is also a method for isolating NSCs and glioma CSCs by culturing these cells in neurospheres [40]–[42], but this method may favor selecting cells that express growth factor receptors, which are not always directly associated with stem cell properties [43]. Some researchers connected CSC phenotype with the increased expression of the cell cycle genes [44],[45] but normal stem cells were shown to infrequently divide generating an actively proliferating cell and a quiescent cell [46]. The actively proliferating cell becomes more and more differentiated with each division, while the quiescent cell retains its stem properties to initiate all types of cells characteristic of a given tissue. One of the reasons behind CSCs resistance to current chemotherapy regimens is the block of their growth and division, since most chemotherapy drugs can affect only actively dividing cells by causing cell cycle arrest [27],[47]. Based on published data, we suggest that quiescent CSCs can be delineated by co-expression of resistance genes to existing therapy (MGMT, APNG, p16INK4A) [48], normal neuronal precursors genes (HES1, TSC22D1, KDM5B, POU3F2, NFIA and NFIB) [49],[50] combined with low activity of the cell cycle genes (IGFBP5, VEGFA, SLC2A3, LGALS3, FAM115C, MT1X, UBC, C4orf3, FAM162A, PPP1R15A, EEF1A1, FTL) [44]. Isolation of this population will allow identifying critical genomic and/or epigenomic glioma CSC determinants responsible for their resistance and the differentiation block.

Despite the significant progress in the study of the molecular, cellular, and tissue-specific processes occurring during malignant transformation, incomplete understanding of molecular mechanisms determining the development of various cellular populations in gliomas complicates the breakthrough in their treatment.

## 3. Glioma molecular profile

The importance of glioma molecular profiling was underscored by the WHO in 2016, when it issued a new glioma classification based on certain mutations in the genes coding for isocitrate dehydrogenases (IDH1, IDH2) and 1p/19q codeletion. Specifically, gliomas are divided into IDH-mutant (IDHmt) and IDH-wild type (IDHwt), IDHmt further subdivided to the ones bearing 1p/19q codeletion and ones with the ATRX (ATP-dependent helicase ATRX, X-linked helicase II) mutation. Thus, tumors carrying mutations in the IDH gene concurrently with 1p/19q codeletion are classified as oligodendrogliomas, while those bearing mutations in IDH and ATRX are referred to as astrocytomas, and IDHwt tumors are identified as primary glioblastomas [51].

The study of the glioma molecular profile also enables predicting the disease development speed and cancer scoring. For example, IDHwt gliomas have the lowest mean overall survival rate [52], while the presence of a specific mutation BRAF V600E is associated with an increased overall survival [53],[54]. Among patients with diffuse gliomas, the best prognosis is associated with IDHmt and 1p/19q codeletion [55],[56] as well as with the ATRX loss of function [55]. Furthermore, the mutation of the TERT gene promoter is unfavorable in IDHwt tumors but beneficial in IDHmt tumors [57]. This indicates the presence of the mutual influence of different mutations. In addition, it was reported that despite the histological identity, primary and secondary glioblastomas have completely different molecular profiles. Thus, 90% of the primary glioblastomas are IDHwt and carry mutations in the genes involved in molecular pathways involving p53, retinoblastoma 1 (Rb1), and tyrosine kinase receptor (RTK/RAS/PI3K) [58]. Secondary glioblastomas are almost invariably IDHmt and carry TP53 and ATRX mutations [59], pointing to their most probable source of origin - low-grade astrocytomas. Meanwhile, the overall survival of patients with primary glioblastoma is about 11 months, while the same index for patients with secondary glioblastoma is more than double (27 months) [60]. Additionally, several studies have separately investigated some heritable genomic variants that could be utilized for glioma risk prognosis, for instance, the mutation in CCDC26 is associated with the oligodendroglioma and astrocytoma development [61],[62], CDKN2B mutation is linked with low-grade astrocytomas; VTI1A, ZBTB16 [63], PHLDB1 [64] rs12230172 (noncoding), and ETFA defects could be considered as risk markers for low-grade IDHmt gliomas; while RTEL1 alteration can be common for all gliomas [65],[66]. Meanwhile, the most morbid IDHwt primary glioblastomas, with the poorest prognoses, could also be predicted in advance using such genomic variants as 3q26.2 near TERC, 7p11.2 near EGFR, and 12q23.33 near POLR3B [63],[65]–[67]. While significant correlations of these genomic variants with the probability of further acquisition of the specific glioma subtypes needs to be addressed in more detail, the aforementioned markers can be clinically assessed in people with family glioma history.

While the role of IDH mutation as a diagnostic marker and predictor of glioma development has been investigated, specific mechanisms determining lower disease grade in the case of this mutation remain unclear. The IDH1 gene encodes isocitrate dehydrogenase, which converts isocitrate to alpha-ketoglutarate in the Krebs Cycle. It was shown that the introduction of mutant IDH1 in human primary astrocytes causes the hypermethylation of histones and DNA sites linked to terminal differentiation, which can lead to locking cell in the embryonic state associated with unlimited self-renewing capacity [68]. This suggests an important relationship between the presence of certain mutations and the epigenetic regulation of transcribed genes. At the same time, mutations in stem cells are considered to be the most malignant, since they are the alleged progenitors of the whole tumor. However, this contradicts the observed lower grade of IDHmt gliomas compared to IDHwt ones. Possible explanations could be either different glioma-initiating progenitor cells for IDHmt and IDHwt glioma types, or distinct secondary epigenetic perturbations of chromatin blocking the final differentiation and determining different transcriptional cellular profiles. In the latter case, glioma CSC molecular phenotyping and tracking their changes in culture should help unravelling the effects of different DNA mutations on tumor fate and delineating factors responsible for the glioma development on case-to-case basis.

## 4. Cell-based glioma model systems

A representative *in vitro* cell glioma cell-based model should mimic the following primary glioma features: i) include defined driver mutations, especially those important for prognosis and diagnosis (IDH, TP53, ATRX, 1p/19 codeletion) and ideally non-canonical alterations (CIC, FUBP1, TERT, NOTCH1, DAXX, EGFR, PTEN, NF1, RB1); ii) resemble epigenetic, transcriptional, and phenotypic profiles of primary tumors; iii) mimic tumor microenvironment including immunocompetent cells that create local immunosuppressive milieu and stromal cells that constitute scaffold for glioma cells [18]; iv) include three-dimensional extracellular matrix creating a special niche glioma cells grow in . We will next discuss cell-based glioma model systems including their relevance including heterogeneity, tumor microenvironment, and extracellular matrix, as well as their putative applications for testing novel chemotherapy regimens and other medical approaches.

### 4.1. Glial cell lines

The first glial cell lines were derived from induced animal gliomas in the 1960's [69]–[71]. It was a breakthrough, as it significantly simplified studies of certain aspects of glial cell biology such as molecular features and signaling pathways (Table 1). However, such cell lines have both advantages and disadvantages as model systems. One major advantage is the unlimited number of cells available for research and the possibility of rapid and reproducible testing of new anti-tumor drugs *in vitro*, as a fast and cost-effective preliminary screening for further testing in appropriate preclinical models. Such tumor cells are convenient to handle, manipulate and maintain in culture, as they are certified to be free of contamination and viral or bacterial infection in contrast to primary glioma cell cultures. The medium for the established glioma cell culture is usually a standard mammalian tissue culture medium supplemented with serum, which is generally available and easy to prepare.

However, recently it was reported that long-term cell culturing leads to genetic drift, chromosomal aberration accumulation and phenotypic alterations, ultimately resulting in poor reproducibility of the data across different laboratories. Over the passages, cell lines acquire new genetic alterations leading to varying phenotypes. U87 was generated in 1960`s from patient with a glioblastoma, but the recently performed whole genome sequencing revealed a great number of indels and translocations, most of which were acquired over decades of cell cultivation [72],[73]. Additional drawback of the standard cell line use is the considerable difference from *in vivo* tumor growth conditions leading to the poor reliability, and making animal models indispensable. However, using animals is a very cumbersome phase in preclinical drug development, often lacking reliability when it comes to clinical trials, and on top of that, may be considered ethically challenged. Good laboratory practice warrants the uniform cell culture, provided it originates from the single origin, but it fails to represent the native tumor microenvironment and interactions between tumor cells and the non-neoplastic environment. Moreover, most cell lines differentiate and reach full maturity, therefore, preventing studies of the CSCs biology [74].

**Table 1.** The most common glioma cell lines.

Oligodendrocyte cell lines			
Cell line name	Method used to generate cell lines		References
CG4 (rat)	Spontaneous immortalization of oligodendrocyte precursors (OPs) from mixed glial culture of neonatal cortex (Sprague–Dawley rat)		[75]
OLN-93 (rat)	Spontaneous immortalization of OPs from mixed glial culture of neonatal brain (Wistar rat)		[76]
Oli-neu (mouse)	Transfection with a t-neu oncogene of primary culture enriched in oligodendrocytes from day 15 embryos (NMRI mouse)		[77]
N19 (mouse) N20.1 (mouse)	Transfection with temperature-sensitive SV40 large T antigen of primary culture enriched in oligodendrocytes from neonatal brain (BALB/cByJ mouse)		[78]
G26-20 (mouse) G26-24 (mouse)	Clones derived from the glioma G26 induced by methylcholanthrene treatment (C57BL/6 mouse)		[70]
HOG (human)	Cells derived from human surgically removed oligodendroglioma		[79]
MO3.13 (human)	Fusion of a human tumor rhabdomyosarcoma RD cell line with a human primary culture of oligodendrocytes from surgery (human)		[80]

Astrocyte cell lines			
Cell line name	Method used to generate cell lines		References
C6 (rat)	Clone derived from a brain tumor induced by N-nitrosomethylurea treatment of adult animals (outbred Wistar rat)		[81]
DI TNC1 (rat)	Transfection with SV40 large T antigen under the control of GFAP promoter of type 1 astrocytes in primary culture isolated from the diencephalon of neonatal brain (Sprague–Dawley rat)		[82]
BALB SFME (mouse)	Clone derived from spontaneous immortalization of day 16 embryonic brains cultured in medium deprived of serum (BALB/c mouse)		[83]
A172 (human)	Clone derived from a surgically removed solid glioblastoma (human)		[84]
U-87MG (human)	Clone derived from a surgically removed malignant astrocytoma grade III (human)		[85]
U-251 (human)	Clone derived from a surgically removed glioblastoma (human)		[86]

### 4.2. Primary glial cell cultures from fresh human material

Recent research in glioma biology has focused on a model system of primary glioma cells derived from freshly resected patient tumors. *In vitro* studies of primary glioma cell cultures from fresh human material are important to identify mechanisms contributing to oncogenesis and tumor growth. However, primary cell cultures are often difficult to prepare and offer only a limited number of cells for research over a limited time span. Moreover, all patients must sign up the informed consent, while all procedures have to be approved by the ethics and legal committees. All these additional steps complicate the establishment of primary glioma cell cultures.

Traditionally, primary tumor cells were extensively passaged and grown in adherent monolayers, therefore, it was impossible to modulate such tumor features as hypoxia, cancer genetic background, and supportive extracellular matrix. It is widely accepted that primary glioma cells should be passaged as little as possible preventing epigenetic or genetic alteration, while cultured without serum. This enables retaining much greater similarity to the primary tumor due to the absence of serum inducing spontaneous CSCs differentiation to maintain their properties [87]. However, the appropriate culture conditions of primary glioma cells do not guarantee the representability of this model. Recently it was shown that adherent cells in classic 2D culture undergo profound phenotypical changes and demonstrate obviously different responses to anticancer drugs than those observed in patients [88]. Moreover, primary monolayer cell cultures fail to demonstrate radiation resistance in contrast to 3D cultures embedded in laminin-rich extracellular matrix [89],[90]. Additionally, it is impossible to reproduce hypoxic conditions in 2D culture, which are essential for selecting the most malignant glioma cells expressing chemo- and radio-resistance genes [91],[92]. However, it is necessary to note that primary glioma cell cultures ensure that the genetic heterogeneity of a given tumor is fully represented [93]. The presence of CSCs in primary glioma cell cultures makes them a convenient research model, as those cells were revealed to have a broad differentiation potential, being able to contribute to the formation of the various tumor subpopulations and tumor neovascularization due to CSCs ability to transdifferentiate into endothelial cells and pericytes [94]–[96]. Additionally, patient-derived tumor models obviously simplify the development of personalized therapy, as it is possible not only to define responders and non-responders for several drug combinations but also to delineate the molecular basis for the acquired drug resistance [93].

### 4.3. Brain tumor spheroids (neurospheres)

The surgically obtained gliomas can be processed to stable cell cultures by maintaining glioma cells as spheroids in serum-free media. Neurosphere assay is a current gold standard for identifying the unique stem cell population in brain tissue [97],[98]. For this purpose, neurosphere culture medium lacks FBS while containing basic fibroblast growth factor (bFGF), epidermal growth factor (EGF), and neuronal viability supplement B27 [87],[99], enabling neural stem cells and glioma CSCs to proliferate and generate multipotent floating cell clusters called neurospheres [97],[98]. The prove of stem properties is achieved by demonstrating that individual neurospheres give rise to neurons, astrocytes and oligodendrocytes after the growth factors removal. However, the neurosphere assay protocols are not uniform and vary significantly between studies. To investigate glioma CSC self-renewal and differentiation potentials these cells should be plated as single cells per well to ensure clonality, i.e. same genetic background in each of the expanded cells within each well. However, researchers prefer to plate multiple cells at a low density to achieve sufficiently high proliferation rate, as paracrine and cell-to-cell signals significantly facilitate cell growth and division. This can lead to difference in the results interpretation and conclusions regarding stem cell properties and corresponding biomarkers. Additionally, it is highly recommended to prove that all spheres arise from cell proliferation (using time-lapse imaging) and are not resulting from mere cell aggregation [43].

Neurosphere assays are more relevant for modelling brain tumor biology then traditional 2D culture conditions. However, the link between this biology and clinical behavior has yet to be established and the success rates of generating neurosphere cultures from gliomas is broadly dependent on tumor grade and genetics. Specifically, generation of cell cultures from IDH-mutated low-grade (WHO grade II and III) gliomas is remarkably difficult, with only few examples reported in the literature [100],[101].

Furthermore, the genetic heterogeneity of human gliomas can result in remarkable variability of CSC properties in patient-derived glioma spheroids depending on the selection of particular cell clones. This immediately raises important questions of how the CSCs diverge into the heterogeneous subpopulations and what exactly are the CSCs. This issue is still not resolved. Currently, OPs and neural stem cells (NSCs) are two main candidates for CSC origin in glioma. This was revealed by the introduction of the same mutations in the OPs and NSCs, that, however, caused the formation of different glioma types [102], thus indicating the interaction between the activated oncogenic molecular pathway and the epigenetic status of the cell at the certain stage of differentiation. On the other hand, the activation of different mutations in the same progenitor cells led to the formation of histologically different gliomas [103]. Thus, the nature of cross-dependences between oncogenic mutations and transcriptional or epigenetic outcomes in brain stem cells in the cases of particular glioma tumors are compelling.

The most important drawback of neurosphere forming assays is their inapplicability for quiescent CSCs detection. Based on the concepts developed for stem cells in healthy tissues, glioma CSCs are believed to divide asymmetrically generating one quiescent cell resistant to chemo- and radiotherapy [104],[105] and responsible for tumor progression [27],[106] and another non-stem-like daughter cell lacking tumorigenic potential [107]. This hypothesis assigns CSCs, and specifically their quiescent subclones, as the most important population for isolation and characterization with respect to their driver mutations as well as epigenetic and transcriptomic signatures. However, quiescent cells may not be capable of forming spheres and it is hard to point to components of the *in vivo* niche required for the activation of dormant stem cells [43].

Nevertheless, lack of glioma CSCs markers and absence of methods for their cultivation do not imply their incompetence to generate tumors *in vivo* [108]. Even though *in vitro* glioma models are promising tools for further elucidation of the molecular and functional characteristics of glioma CSCs (including identification of the best markers for recognizing these cells) [109], so far this has not led to broad consensus on what exactly glioma CSCs are and how they impact on the disease progression.

### 4.4. Towards the innovative model system

While the influence of individual components on the emergence and growth of gliomas was extensively explored, studies evaluating several cancer-driving factors simultaneously are rare. Predominantly researchers focus on finding mutations causing the development of the malignant processes, yet often missing the effect of concomitant mutations, transcriptional profiles, epigenetic regulators and microenvironment of the tumor cells [110]. This warrants the increasing pressure to develop new *in vitro* model systems allowing to monitor dynamic changes in condition resembling *in vivo* development of the tumor cell poulations, e.g. CSCs.

Tumor progression and invasion are closely coordinated with the microenvironment, which includes a network of various cell types, e.g. stroma, blood vessels, secreted factors, and surrounding matrix. Astrocytes are considered the most abundant glial cells in glioma microenvironment and therefore significantly contribute to the glioma pathogenesis. They surround and infiltrate glioma cells and protect them from cytotoxic effects caused by various anti-tumor drugs [111],[112]. What is more, astrocytes could increase the IL-6 secretion and stimulate the expression of membrane type-1 matrix metalloproteinase (MT1-MMP or MMP14), a proteolytic enzyme known to be involved in extracellular matrix degradation and assist cancer invasion and progression [113].

The majority of the non-neoplastic cells in gliomas are tumor-associated macrophages either of peripheral origin (monocytes) or representing brain intrinsic microglia. They produce a variety of stromal factors and cytokines to create supportive stroma for tumor expansion [114],[115]. Furthermore, tumor-associated macrophages perform immune functions. In this manner, tumor-associated macrophages facilitate tumor proliferation, survival, and migration [116]. T- and B- lymphocyte infiltration also occurs in glioma and the presence of tumor-infiltrating lymphocytes (TILs) is predictive of a clinical outcome [117], as they also play a key role in immune response.

The immunosuppression in glioma occurs due to the increased synthesis of immunosuppressive cytokines (IL-6, IL-10, TGF-β and PGE2), as well as tumor-promoting cytokines (IL-1 and bFGF), inhibition of T-cell proliferation and effector responses, activation of Tregs, and suppression of NK-cell activity [118]–[120]. The glioma microenvironment also promotes immunosuppression by increasing T-cell apoptosis through cooperative interaction between CD70 and gangliosides [121]–[122].

Gliomas are highly vascular tumors. Development of the brain tumor vascular niche, (perivascular niche) environment requires bone marrow derived cells. Among them are endothelial progenitor cells that mature into endothelia of the vessels; pericyte progenitor cells that surround the vasculature and mature into pericytes and vascular smooth muscle cells [123]. The vascular system in gliomas is characterized by endothelial hyperplasia and microvascular proliferation [124]. The latter represents the regions of angiogenesis, which is crucial for glioma progression. These angiogenic regions including the perivascular niche are considered crucial for CSCs maintenance [125]–[127].

Therefore, aforementioned complex cell-to-cell interactions establish a unique glioma tumor ecosystem. Thus, inclusion of the cells, that constitute glioma microenvironment into an *in vitro* model significantly changes signaling within tumors [128]. Moreover, the heterogeneous tumor environment influences on cell proliferation rates, produces the regions of hypoxia and acidity, as well as it regulates the malignant cell transformation and the sensitivity to chemotherapy [129].

One of the possibilities to conjointly investigate tumor microenvironment associated features is culturing CSCs in hydrogel scaffolds, that can maintain them, allowing for supplements and mimicking extracellular matrix (ECM) conditions. Culturing in hydrogels also confers cells with the ability to receive signals not just at their ventral surface but in all three dimensions as it occurs normally *in vivo* [130].

Hydrogel is a water-swollen material which is composed of cross-linked polymer (e.g. polysaccharide) chains of natural or synthetic origin sometimes including protein or other molecules. They possess the similarity to the natural tissue due to significant water content, and therefore can serve as highly effective matrices for 3D cell cultures [131]. Natural hydrogels for cell culture generally consist of proteins and extracellular matrix compounds such as laminin, hyaluronic acid, collagen, or fibrin. Since such hydrogels are isolated from natural sources, they are inherently biocompatible and bioactive [132]. Collagen I is a common extracellular matrix molecule, which is found in stroma, can be derived from rat tail tendon, bovine skin, or human placenta. Notably collagen I interacts with integrin receptors to modulate the gene expression [133]. Among target genes there are those that adjust the production of matrix metalloproteinases enzymes, and those that influence on cell proliferation, cell migration [134] and cell sensitivity to anti-cancer treatment [135]. Hydrogels containing substances imitating developing brain extracellular matrix, such as collagens type I and IV or artificial fibroins, were proven to effectively induce the NSC differentiation [136], or conversely, embryonic stem cells can retain their pluripotency upon continuous cultivation in hydrogels containing glycosaminoglycans with varied esterification state [137]. Additionally, commercially-available Corning Matrigel matrix, is widely used for 3D modeling of cancer tissue culture. It is derived from the reconstituted basement membrane tissue from the mouse Englebreth-Holm-Swarm tumor [138] and is considered to contain various extracellular matrix components, which switch several signaling pathways in cancer cells responsible for cancer cell motility [139], angiogenesis [140], and drug sensitivity [141]. However, the representative power of such models in tests involving human cancer tissue and inherent batch-to-batch variability of such matrices are still questionable. Hydrogels are used as standalone 3D matrices or are associated with solid scaffolds, permeable supports, cellular microarrays, and microfluidics devices. Additional support for hydrogel model over the conventional 2D-culture is provided by greater resistance to chemotherapeutics in cells grown in collagen-rich stiff hydrogels, thus underscoring the high amenability of such models for the reliable drugs prescreening [142].

In order to mimic the *in vivo* microenvironment, 3D *in vitro* models without scaffolds have been produced, which are named as spheroid model. Spheroid model is a heterogeneous aggregate of various cells not attached to an external surface for support, providing more biologically relevant data than 2D monolayers due to the natural cell-to-cell interactions and the appearance of hypoxic and necrotic regions [143].

One attractive possibility to conjointly investigate several tumor associated conditions is to adapting an aggregate culture model to glioma, i.e. to arrange sorted CSCs together with stromal, immune, and endothelial cells and then as aggregates on a filter floating in a culture medium [144]–[146]. This model will encompass such important *in vivo* tumor features as local hypoxia, cytokines produced by the immune cells, stromal cells constituting the scaffold, and resulting 3D-structure enabling native cell-to-cell interactions. Proof-of-principal experiments were conducted for pancreatic progenitor cells, and it was shown that they can differentiate into mature endocrine cells both within hanging drop and on floating filter [147]. As for 3D cancer co-culture models, they were created for a variety of cancer types (Table 2).

**Table 2.** Cancer co-culture models.

3D models	Culture matrix	Reference
Breast epithelial cells co-cultivated with endothelial cells. This 3D model was designed to study the role of endothelial cells in the development and growth of normal and cancerous breast epithelial cells	Transwell® filters (0.4 µm); Corning® Matrigel® matrix	[148]
Mouse breast cancer cell line co-cultivated with stromal fibroblasts. This model was developed to study the effects of stromal cells on breast cancer progression	Corning Matrigel matrix	[149]
Ovarian cancer cells co-cultivated with mesenchymal stem cells. This model was created to study the influence of mesenchymal stem cells on ovarian cancer cells migration and invasion in the amniochorionic membrane model	Transwell filters; Ultra-Low Attachment plates; Corning Matrigel matrix; Amniotic membrane scaffold	[150]
Melanoma cells co-cultivated with keratinocytes, dermal stem cells and melanocytes. This 3D model was constructed to study the melanoma progression	Bovine Collagen I	[151]
Melanoma cells co-cultivated with keratinocytes. Melanoma spheroid model for the *in vitro* drug testing	Collagen I	[152]

3D co-culture models are especially appropriate for studying cell-to-cell interactions, and allow studying normal cells contribution to tumor growth, vascularization, and metastasis. It was confirmed that stromal cells activate chemo-resistance and protect tumor cells from the anti-cancer drugs toxicity [149]. Endothelial cells contribute to blood supply permitting tumor growth and survival [153]. Moreover, endothelial cells can sensitize tumors to the chemotherapeutic drugs *in vitro*, and can stimulates angiogenesis and metastasis *in vivo* [154].

Unfortunately, so far there were no satisfactory 3D cancer co-culture models for glioma cancer cells reported to date. We suggest tumors should be divided into single cells and sorted. In order to culture CSCs on floating filter for further examination Sorted cells may be mixed with cultured tumor stromal and immune cells and then cultured within hanging drop for 24h to form spheres. Spheres may be cultured on the floating filter for days, in the dish containing serum-free medium. Changes of CSC markers expression could be monitored by immunohistochemistry FACS, mass spetrometry, or real-time PCR [147].

Moreover, there is a possibility to simulate poorly-infiltrated tumor models by adding lymphocytes at a low density by adding low dose of special immunocytokine (DP47-IL2v, 10 nM) promoting lymphocyte proliferation and infiltration into pre-formed aggregates, yielding highly-infiltrated tumor models [145]. Furthermore, it was reported that in 3D melanoma co-cultures, cancer cells inhibited T-cells activation and proliferation, demonstrating that spheroids support a more physiological immune-modulatory function than monolayer adherent cultures [155]. Tumor 3D homotypic spheroids, composed of tumor cells, fibroblasts, endothelial cells, and immune effector cells, were also referred to as a more appropriate model [156]. Using such system, it is possible to simultaneously simulate tumor microenvironment by constructing the 3D structure, creating hypoxia zones within the aggregates and the native ratio between immune and stromal cells. This system will be highly applicable for tracing CSC differentiation pathways depending on the microenvironment and the framework component of the matrix. Appearance of the more committed progenitors of glioma CSCs will be confirmed by the attenuation of embryonic (Oct4, Sox2, Nestin) and adult stem cell markers (CD133, CD44, CD15), and upregulation of the progenitor cell markers (PSA-NCAM and Doublecortin) and oligodendrocyte terminal differentiation markers of (ASCL1, OLIG2, DLL3), astrocytes (GFAP), neurons (β-tubulin III, SYT1 and SLC12A5), and markers of inflamed astroglial cells (SERPINE1, TGFB1, RELB). In addition, the absence of normal differentiation markers for neurons and neuroglia, NCAM1 and Sox9, respectively, has to be confirmed. Moreover, the study of transcriptomic, proteomic and epigenetic markers of a certain GSC differentiation stage will enable to suggest the ways inducing GSC differentiation, which can be utilized for differentiation therapy successfully applied for the treatment of acute promyelocytic leukemia [157], but not yet properly developed for the glioma therapy [158].

Although the systems described above are already relatively complex when compared to standard cell line culture, the culture models are constantly evolving in attempt to match the complexity of native tissues. The approaches discussed here, including possible systems for CSC culturing, assume finely tunable properties leading to defined response from resident cells, and therefore promise to facilitate the design of representative cell models in the future.

## 5 Conclusion

Currently there is a lack of satisfactory methods for detailed monitoring of molecular and cellular determinants of tumor growth in patients as well as ideal *in vitro* systems for modeling of these processes. The innovative model systems should mimic such tumor features as hypoxia, native tumor microenvironment, and three-dimensional extracellular matrix. The reliability of the models should be validated on genomic, transcriptomic, proteomic, and epigenetic levels in comparison with the corresponding primary tumors characteristics. The model systems should simplify the investigation of the effect of different culturing types on differentiation and proliferative activity of glioma CSCs. Furthermore, the development of representative, and generally accessible *in vitro* model of glioma CSCs allowing investigator to control its differentiation, will facilitate their research, as well as the screening of the prospective anti-tumor drugs and/or alternative treatment approaches.
